# Characteristics of Maxillary Sinus Septa: A Cone-Beam Computed Tomography Evaluation

**DOI:** 10.1155/2022/2050257

**Published:** 2022-10-05

**Authors:** Ahmad Assari, Najwa Alotaibi, Maha A. Alajaji, Amal Alqarni, Maha Ali Alarishi

**Affiliations:** ^1^Oral and Maxillofacial Surgery and Diagnostic Sciences Department, Riyadh Elm University, Riyadh, Saudi Arabia; ^2^Ministry of Health, Second Health Cluster, Riyadh, Saudi Arabia; ^3^Alajaji Polyclinic, Riyadh, Saudi Arabia; ^4^Ministry of Health, Jazan, Saudi Arabia

## Abstract

**Objective:**

Our study aimed to determine the prevalence, location, and morphological differences of the septa using CBCT images.

**Methods:**

This retrospective study was conducted on CBCT examinations. The sample included both mixed and permanent dentition and edentulous patients. The images were viewed in 3 planes (sagittal, coronal, and axial) and the left and right maxillary sinuses were evaluated for the septa's prevalence, location, and morphological differences.

**Results:**

The measurements were statistically analyzed using SPSS software. Out of 200, 122 patients showed one or more bony septa in the maxillary sinus. The septal location and angulation were not limited to a specific area or a specific range. Significant differences between genders were found.

**Conclusions:**

The increased surface area of the septa using plane 2-dimensional radiographs is impossible. CBCT has improved the planning of any sinus procedure and offers adequate information compared to conventional radiographs.

## 1. Introduction

Maxillary sinus septa are thin walls of cortical bone projecting from the sinus floor. Underwood's anatomical study first described them [1]. Moreover, the irregular resorption pattern of the maxillary alveolar process leads to the formation of bony crests on the sinus floor [2]. Krennmair classified septa into primary and secondary septa, where the first arises during the development of the maxilla and the latter arises following irregular pneumatization of the sinus [3]. The prevalence of the septa ranges from 10% to 58% in the literature. They were considered clinically insignificant until surgical complications following endoscopic and augmentation for the maxillary sinus were reported [4–6].

Sinus augmentation procedures have been routinely performed to allow anchorage of dental implants even with severe cases of ridge resorption [7], and the increased demands for replacing missing teeth have led to an increased number of sinus surgical procedures by general practitioners. The lack of training and ignorance of anatomy are important factors in the success of surgical treatment [8]. Schneidarian membrane perforation is the most commonly reported complication following sinus elevation procedures. Tearing of this membrane is often associated with the presence of maxillary sinus septa [9]. In order to achieve a successful sinus augmentation and to avoid any complications during the procedure, it is critical to evaluate the septa before surgery for a successful outcome [10].

Farronato et al. [11] conducted a study to test the validity of a novel protocol for 3D sagittal jaw discrepancy assessment (skeletal class determination). The study found that, without using *S* and *N* ceph-alometric landmarks, AF-BF demonstrated great reliability in skeletal class determination on limited FOV CBCT. Comparing the traditional 2D indexes and using a smaller field of view (10 10), CBCT at least shows the Frankfurt plane to the *B* point vertically and the most anterior between the *A* and *B* point to the Po point horizontally.

Despite the interindividual heterogeneity of their morphology, a previous investigation assessing the association between the frontal sinus shape and facial growth pattern found a correlation between frontal sinus dimensions and craniofacial characteristics [12]. While vertical growth is still occurring in young individuals whose frontal sinuses have achieved their maximum size, a larger frontal sinus may indicate future vertical growth.

Different radiological methods have been used to evaluate the sinus septa. CBCT has the appropriate resolution to identify and visualize osseous details in the maxillary sinus, in addition to its advantages biologically compared to computed tomography [9]. The objective of our study is to determine the septa's prevalence, location, and morphological differences using cone-beam computed tomography (CBCT) images.

## 2. Materials and Methods

**Figure 1:**
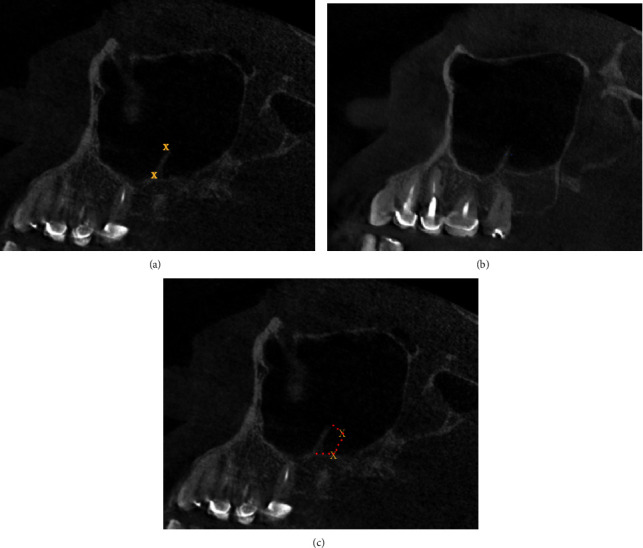
CBCT cuts showing the four points used in the calculation. Two medial points (blue) and two lateral points (blue).

This retrospective study was conducted on CBCT examinations taken from patients who attended the oral diagnostic department at Riyadh College of Dentistry and Pharmacy, University of Riyadh, Saudi Arabia, between 2011–2015. Images were obtained in Sirona Galileos (Sirona Dentsply, Bensheim, Hessen, Germany) at 85 kV, 5–7 mA, and 14 s. This study was approved by the Institutional Review Board of RCsDP (RC/IRB/2016/004). It comprised 200 patients who were divided into Group *A* (<30 years), Group *B* (31–45 years) and Group *C* (>45 years). Images of patients who received a dental implant or had a sinus surgery history were excluded. Four examiners evaluated 400 maxillary sinuses for the location of two anatomical points on three-dimensional aspects (axial, coronal, and sagittal). Images were exported and were viewed using a digital image analysis software (KDIS 3D imaging software 1.4_Carestream Health Inc.), which allowed the measurement of the landmarks on three planes (axial, sagittal, and coronal) on 287 *μ*m cuts. The number of septa in both maxillary sinuses was recorded. Each septum was evaluated as a bony plate, where four points were measured for each plate (superior lateral, superior medial, inferior lateral, and inferior medial) in axial, coronal, and sagittal images. These points allowed calculation of the septa surface area (Figure 1).

According to ANS-PNS, each maxillary sinus bearing a septum was divided into three portions vertically and three horizontally. The distance from the anterior part of each septum to the coronal level of ANS was measured and considered as horizontal relation. The distance from the most inferior part of each septum to the level of the ANS-PNS line was measured and considered as vertical relation. The angulation of the septa plate was mathematically measured by calculating the plate's average inclination to a line from ANS-PNS. All measures were obtained using the coordinate methods.

### 2.1. Statistical Analysis

The SPSS version 23 was used to analyze the data. The frequency and percentages were calculated for categorical variables, and mean and standard deviation was computed for quantitative variables. The *T*-test was applied to observe the mean difference between genders. ANOVA was applied to observe the mean difference between age groups, and Pearson correlation was applied to observe the correlation. A *P* value <0.05 was considered to be significant.

### 2.2. Reliability and Assessment

For inter-examiner reliability, the 4 examiners evaluated 30 scans, and their results were calculated using Cronbach's alpha for each point. The average Cronbach's alpha for the points was found to be 0.74.

## 3. Results

Out of the 200 samples, 188 samples were analyzed, and 12 cases were not included due to missing data. Female dominance 118 (63%) and age group >45 years were more prevalent in this study, 85 of the sample (45%). Septa were evaluated for their location, surface area and angulation. Out of 188, 135 (72%) patients showed the presence of at least one septum which 71 (38%) showed the presence of a single septum, 83 (44%) showed two septa, 24 (13%) showed three septa, and 10 (5%) showed four septa. The mean surface area, horizontal relation, vertical relation and angle is presented in Table1. *T*-test was applied to observe the association of the mean surface area, horizontal relation, vertical relation, and angle with gender. Only the vertical relation of females was greater than males. It was found statistically significant, as shown in Table 2. ANOVA was applied to observe the mean difference of the surface area, horizontal relation, vertical relation, and angle between 03 age groups and a statistically nonsignificant difference was observed. Pearson correlation was applied, and the results are presented in Table 3.

## 4. Discussion

To our knowledge, we evaluated the prevalence of maxillary sinus septa in 188 samples. This is the first radiological study evaluating the surface area of sinus septa. The surface area of the septa ranged from 5 mm^2^ to 388 mm^2^ in this sample. This size of a bony plate may play a major role in the success of sinus surgery procedures. A larger surface area favoured the later inserted graft grafting procedure through a rise in the implant size in terms of dimensions and the number of threads leading to a decline in the transmission of strain to surrounding bone subtitles [13]. A previous study by Schwarz et al. [14] reported an increased risk of perforation rate of the sinus membrane when residual bone height in sinus septa appears to be less than 3.5 mm. The prevalence of septa in our study was 72%, relatively high regarding other reports, which were in the range of 25–35.5%.1, 3, 6. According to this study, the occurrence of the septa within the maxillary sinus was not limited to a specific location or age group. Still, the difference was found when compared between genders. According to a study conducted by Shibli et al. [6], the prevalence of septa was not associated with age; contrary to our findings, it was also not associated with gender. However, Lee et al. [15] found that the septa prevalence was higher in males than females, contrary to our findings. Van Zyl et al. [8] reported the same findings while evaluating the septa location in regards to the teeth. They found that the occurrence of septa was 13% in the anterior region, 25% in the middle region and 15% in the posterior region in their study of 200 patients. The growth of the maxillary sinuses appears to resemble the distinct growth peaks in male and female subjects. In female subjects, sinus development begins at a young age. Sinus growth primarily occurs between the second and third age groups in male subjects, while it begins between the first and second age groups in female subjects and continues between the second and last. During its growing phase, the sinus has a vertical development, which primarily accounts for its volume increase [16].

Naitoh et al. [17] calculated the septum's angle between the anterior maxillary sinus and transverse palatine suture regions. They found that most of the septa in the anterior maxillary sinus region were antero-laterally directed from the interior wall, and most of the septa in the transverse palatine suture region were laterally directed from the interior wall. Munetaka et al. [9] reported an angulation ranging from 49.8 to 127.3 degrees, with a mean of 101.8 degrees. Our study evaluated the angle between the septum and the ANS-PNS line. The angulation of the septum ranged from 0° to 175.49° with a mean of 82.44°, giving a wide range and variation of septa shape among the antrum. Schneidarian membrane perforation is one of the most common complications following a left sinus procedure. Studies showed that septa interfering with that procedure were one of the factors increasing this perforation risk [18, 19]. To avoid such complications, practitioners must be aware of the anatomical environment before any surgical procedure. The increased septa sizes found in this study and their wide distribution in their location within the sinus may increase the incidence of sinus surgeries complication. The presence of the sinus can be evaluated with the conventional panoramic radiological examination, but these plane x-rays cannot show the full depth of the sinus. CBCT has been considered a valuable tool for evaluating objects in the maxillofacial area in three dimensions. Studies have found that it is an accurate and reliable evaluation compared to conventional computed tomography (CT) or even cadaveric assessment. It also has a biological advantage over CTs with a markedly decreased radiation dose. [10] We found that the features of CBCT of generating three planes (axial, coronal, and sagittal) can be easily implemented in mathematical equations and increases the accuracy of measuring different landmarks of interest.

## 5. Conclusion

According to the results of this study, the prevalence of maxillary sinuses with septa was high (72%), and no significant difference was observed among the three age groups. Our study suggests that clinicians should evaluate the maxillary sinus thoroughly to assess the septa during pre-surgical planning, as its occurrence is not limited to one area. More investigations are required to understand the distribution patterns and causes of the maxillary sinus septa. The size of the sinus septa plays a major role in the success of sinus surgeries. Such large septa as presented must be evaluated before any intervention. CBCT has improved the planning of any sinus procedure and offers adequate information compared to conventional radiographs. It is a reliable, accurate tool for examining the size, location, and angulation of such bony plates in the antrum.

## Figures and Tables

**Table 1:** Mean value of the surface area, horizontal relation, vertical relation, and angle.

	Area (mm)	Horizontal relation (mm)	Vertical relation	Angle
Mean	69.5^2^	27.6	8.8 mm	82.4°
Minimum	5.0^2^	12.0	−15.5 mm	0
Maximum	388.3^2^	53.6	51.9 mm	175.4°

**Table 2:** Mean differences in the surface area, horizontal relation, vertical relation, and angle between gender.

		*t* statistics	*P* value	Mean difference	95% confidence interval of the difference	
Group					Lower	Upper
Male and female						

	Surface area	−0.8	0.4	−7.5	−25.7	10.7
	Vertical relation	3.3	0.0	5.1	2.1	8.0
	Horizontal relation	−1.0	0.3	−1.5	−4.5	1.4
	Angle	1.0	0.3	4.5	−4.2	13.3

**Table 3:** Correlation of variables.

		Surface area	Horizontal relation	Vertical relation	Angle
Surface area	Pearson correlation	1	−0.1	−0.002	**0.004**
	Sig. (2-tailed)		0.1	0.9	**0.9**

Age group	Pearson correlation	0.007	0.026	−0.02	**0.059**
	Sig. (2-tailed)	0.9	0.7	0.7	**0.4**

Horizontal relation	Pearson correlation	−0.1	1	−0.5	**0.5**
	Sig. (2-tailed)	0.1		0	**0**

Vertical relation	Pearson correlation	−0.002	−0.5	1	−0**.3**
	Sig. (2-tailed)	0.9	0		**0**

Angle	Pearson correlation	0.004	0.5	−0.3	**1**
	Sig. (2-tailed)	**0.9**	**0**	**0**	

## Data Availability

All the study data are available on request.
